# Body composition analysis using CT and MRI: intra-individual intermodal comparison of muscle mass and myosteatosis

**DOI:** 10.1038/s41598-020-68797-3

**Published:** 2020-07-16

**Authors:** Anton Faron, Alois M. Sprinkart, Daniel L. R. Kuetting, Andreas Feisst, Alexander Isaak, Christoph Endler, Johannes Chang, Sebastian Nowak, Wolfgang Block, Daniel Thomas, Ulrike Attenberger, Julian A. Luetkens

**Affiliations:** 1 Department of Radiology and Quantitative Imaging Lab Bonn (QILaB), University Hospital Bonn, Venusberg-Campus 1, 53127 Bonn, Germany; 2 Department of Internal Medicine I, University Hospital Bonn, Venusberg-Campus 1, 53127 Bonn, Germany

**Keywords:** Biomarkers, Muscle

## Abstract

Computed tomography (CT) and magnetic resonance imaging (MRI) can quantify muscle mass and quality. However, it is still unclear if CT and MRI derived measurements can be used interchangeable. In this prospective study, fifty consecutive participants of a cancer screening program underwent same day low-dose chest CT and MRI. Cross-sectional areas (CSA) of the paraspinal skeletal muscles were obtained. CT and MRI muscle fat infiltration (MFI) were assessed by mean radiodensity in Hounsfield units (HU) and proton density fat fraction (MRI^PDFF^), respectively. CSA and MFI were highly correlated between CT and MRI (CSA: r = 0.93, P < 0.001; MFI: r = − 0.90, P < 0.001). Mean CSA was higher in CT compared to MRI (46.6cm^2^ versus 43.0cm^2^; P = 0.05) without significance. Based on MRI^PDFF^, a linear regression model was established to directly estimate skeletal muscle fat content from CT. Bland–Altman plots showed a difference between measurements of − 0.5 cm^2^ to 7.6 cm^2^ and − 4.2% to 2.4% regarding measurements of CSA and MFI, respectively. In conclusion, the provided results indicate interchangeability of CT and MRI derived imaging biomarkers of skeletal muscle quantity and quality. Comparable to MRI^PDFF^, skeletal muscle fat content can be quantified from CT, which might have an impact of analyses in larger cohort studies, particularly in sarcopenia patients.

## Introduction

Decrease in skeletal muscle quantity and quality, commonly termed sarcopenia, is known as a strong risk factor for adverse outcomes in several chronic and malignant diseases. Sarcopenia was shown to have high socio-economic and personal burden and leads to impaired activity in daily life, decreased mobility, loss of independency and a higher mortality^1–3^. Initially, sarcopenia was considered to be an age-related phenomenon^4^. However, it is now increasingly realized that sarcopenia may also occur in younger patients, for example secondary due to systemic diseases. Moreover, it was realized that sarcopenia may not be captured by conventional anthropometric measurements such as body mass index (BMI) or waist-to-hip-ratio, particularly in obese patients^1,5^.

Amount and quality of skeletal muscles can be assessed by cross-sectional imaging modalities such as computed tomography (CT) and magnetic resonance imaging (MRI). Previous studies indicate that both modalities may provide imaging based quantitative biomarkers of sarcopenia, and that these biomarkers may reveal prognostic information in various severe diseases^6–10^. Thereby, cross sectional areas (CSA) of skeletal muscles at distinct anatomical landmarks were shown to provide accurate surrogates of total skeletal muscle amount and therefore may be used to identify patients with low muscle mass^1,6,7^. The most common approaches to estimate skeletal muscle amount are determination of circumferential skeletal muscle area or psoas muscle area, both typically obtained at lumbar vertebral levels^1^. However, these landmarks are frequently not captured in several imaging protocols, although sarcopenia is known to be a relevant factor in many chronic diseases. Therefore, alternative landmarks were proposed, such as the level of the superior mesenteric artery (SMA)^6^. CT and MRI derived measurements of skeletal muscle fat infiltration (MFI) as indicators of muscle quality were shown to predict survival following transcatheter aortic valve replacement due to aortic stenosis or local ablative treatment of colorectal liver cancer^11,12^. According to international guidelines, functional tests such as grip strength measurements or the chair stand test may be used to identify patients with probable sarcopenia. The diagnosis is than confirmed by determination of low skeletal muscle quantity or quality, for which—as stated in these guidelines—both CT and MRI based measurements may be applied^1,5^.

However, it is unclear how CT and MRI derived measurements of skeletal muscles are related to one another and if obtained measurements of skeletal muscle mass and quality are interchangeable between imaging modalities. Therefore, we aimed to (a) assess CSA and MFI measurements as surrogates of skeletal muscle mass and quality in CT and MRI in subjects who received both imaging modalities at the same day and (b) to compare measurements intra-individually to assess agreement between modalities for determination of skeletal muscle mass and quality.

## Methods

### Study design and population

In this study, a subset of participants of a lung cancer screening program of the University Hospital of Bonn was prospectively enrolled. Consecutive participants who underwent screening between 05/2019 and 08/2019 were included. For each included subject, the individual CT and MRI scans of the chest were performed within the same day. Exclusion criteria were self-reported or validated history of cancer, as well as any type of metallic implants which preclude MRI examination such as cardiac pacemakers, implantable defibrillators, neurostimulators, or cochlear implants. Furthermore, pregnant subjects as well as those with claustrophobia were excluded.

### Imaging protocol

For each study subject, CT and MRI examinations were performed on the same day. Low-dose helical CT of the chest in supine positioning without administration of iodinated contrast was performed on a clinical CT-scanner (Brilliance iCT SP 128 CT, Philips Healthcare, Best, the Netherlands) with the following imaging parameters: tube current (exposure time product), 25 mAs; tube voltage, 120 kV; collimation, 16 × 0.63 mm; reconstructed slice thickness, 2 mm. As it was done in previous studies, a slice thickness of 5 mm was secondary reconstructed to perform muscle measurements^8^.

MRI was performed on a clinical whole body 1.5 T scanner (Ingenia 1.5 T, Philips Healthcare, Best, the Netherlands) using a 32-channel torso coil with digital inter-face for signal reception. The imaging protocol included an axial six-echo 3D spoiled gradient-echo Dixon sequence for chemical-shift encoded fat–water separation with T2* correction. The imaging parameters were as follows: repetition time/echo time (TE)/ΔTE, 7.8/1.08/1.1 ms; field of view, 350 × 302 × 150 mm^3^; acquired voxel size, 1.99 × 1.99 × 6.00 mm^3^; reconstructed voxel size, 0.99 × 0.99 × 3.00 mm^3^; flip angle, 5°. Data were acquired during one breathhold (scan duration: 14.9 s). Proton density fat fraction (PDFF) maps for determination of muscle fat content were reconstructed directly on the imager console after image acquisition using vendor specific software.

### Image analysis

For comparison of skeletal muscle measurements, single-slice images at the level of the superior mesenteric artery (SMA) were exported from CT and MRI image datasets for each subject to a separate workstation. The SMA level was chosen as the anatomical landmark for skeletal muscle measurements since it was previously demonstrated that single-slice measurements of paraspinal skeletal muscles at this level provide a highly accurate surrogate of skeletal muscle mass and may reveal prognostic information in patients with chronic and malignant diseases^6,11,13^. For skeletal muscle analysis, a previously described in-house tool written in MATLAB (The Mathworks, Natick, MA) was adapted to the particular task of this study^6,12,14^. All measurements were performed by a board-certified radiologist experienced in abdominal diagnostic imaging and body composition analysis. Intrareader reproducibility was excellent for assessment of skeletal muscle area [ICC = 0.996; 95% confidence interval (CI), 0.979–0.999; P < 0.001] and skeletal muscle fat infiltration (ICC = 0.996; 95% CI, 0.978–0.999; P < 0.001). Patient information was blinded. Skeletal muscle area was defined as the bilateral CSA of the paraspinal skeletal muscle compartment, including the erector spinae muscles and the spinotransverse muscle group. Due to their low extent and inconsistent expression at the SMA level, the psoas major and quadratus lumborum muscles were excluded from the segmentation. The paraspinal skeletal muscle compartment was identified and its contours were manually traced, separating it from the erector spinae aponeurosis, interspinous ligaments, adjacent parts of the vertebral bodies as well as bordering anatomical structures of the thoracic wall^15^. To allow for highly accurate segmentation, images were displayed in standard display settings with the option to manually adjust contrast between skeletal muscles and adjacent tissues by the reader, where appropriate. For determination of MFI, the mean radiation attenuation in Hounsfield units (HU) and the tissue fat content expressed as MRI^PDFF^ in percent (%) were calculated for the CSA in CT and MRI, respectively.

### Statistical analysis

Statistical analysis was conducted using commercially available statistical software (Prism version 8, GraphPad, La Jolla, CA). Continuous and categorical variables are given as means with standard deviation and total numbers with percentages, respectively. The Mann–Whitney U test was used for group comparison statistics. Continuous data were plotted as violin plots. Intraclass correlation coefficient (ICC) was calculated to assess intrareader reproducibility. ICC estimates and their 95% confident intervals (CI) were assessed for a single rater, based on assessment of absolute-agreement using a two-way mixed-effects model. Pearson correlation coefficients (r) and Bland–Altman plots with mean absolute differences and 95% limits of agreement were calculated to assess interrelations between CT and MRI derived measures of skeletal muscle mass (CSA) and skeletal muscle quality (MFI). Thereby, measurements for assessment of MFI systematically differed between CT and MRI for methodological reasons. While in CT, mean skeletal muscle radiation attenuation is measured to determine fat content of skeletal muscles^16^, MRI^PDFF^ allows for direct assessment of the tissue fat fraction. This disparity precluded direct comparison of established CT and MRI derived MFI measures with the Bland–Altman method. However, as detailed in the results section, CT and MRI derived MFI values were highly correlated with one another. This relation allowed for calculation of a linear regression model for direct estimation of skeletal muscle fat content from skeletal muscle radiation attenuation values based on the corresponding MRI^PDFF^ values. This measure was termed CT^FF^, enabling for direct comparison of CT and MRI derived measures of skeletal muscle fat content on a ratio scale. For all analyses, a P value < 0.05 was considered to indicate a statistically significant difference.

### Ethical approval and informed consent

The presented study was approved by the institutional review board of the University of Bonn and hence all methods were performed in compliance with the ethical standards set in the 1964 Declaration of Helsinki as well as its later amendments. Written informed consent was obtained prior to examination from all subjects.

## Results

### General characteristics

Fifty patients (19 female, 31 male) with a mean age of 61 ± 6 years were evaluated. Baseline characteristics of the study population are detailed in Table 1.

**Table 1:** Baseline clinical characteristics including self-reported history of currently treated chronic diseases of the study population.

Variable	All subjects (N = 50)
Age (years)	61 ± 6
Body height (cm)	173 ± 8
Body weight (kg)	80 ± 16
Body mass index (kg/m^2^)	27 ± 4
Diabetes	5 (10%)
Hypertension	17 (34%)
Dyslipidemia	8 (16%)
Chronic obstructive pulmonary disease	5 (10%)
Coronary artery disease	2 (4%)
Asthma	2 (4%)

Continuous variables are expressed as means with standard deviation. Categorical data are given as total count as well as percentages.

Mean cross-sectional area (CSA) of the paraspinal skeletal muscles was significantly higher in male patients compared to female patients in both CT (51.8 ± 10.5 cm^2^ vs. 38 ± 4.8 cm^2^, P < 0.001) and MRI (47.4 ± 11.7 cm^2^ vs. 35.8 ± 4.1 cm^2^, P < 0.001). For the entire population, mean CSA tended to be higher in CT compared to MRI (46.6 ± 11 vs. 43.0 ± 11.1 cm^2^, P = 0.050). No significant differences between area-based measurements obtained for the left and right paraspinal skeletal muscle compartments were observed for both male and female patients (Table 2, all P > 0.05).

**Table 2:** Assessment of skeletal muscle area.

Modality		All subjects (N = 50)	Male (N = 31)	Female (N = 19)	P-value
CT	Total (cm^2^)	46.6 ± 11.0	51.8 ± 10.5	38.0 ± 4.8	< 0.001
	Right (cm^2^)	23.7 ± 5.4	26.4 ± 4.9	19.2 ± 2.1	
	Left (cm^2^)	22.9 ± 5.8	25.5 ± 5.7	18.8 ± 3.0	
	P-value		0.401	0.525	
MRI	Total (cm^2^)	43.0 ± 11.1	47.4 ± 11.7	35.8 ± 4.1	< 0.001
	Right (cm^2^)	21.7 ± 5.7	23.9 ± 6.0	18.0 ± 2.4	
	Left (cm^2^)	21.3 ± 5.6	23.5 ± 6.0	17.8 ± 2.1	
	P-value		0.925	0.628	

Cross-sectional areas of the paraspinal compartment are given as bilateral (total) skeletal muscle area as well as the respective right and left compartments in cm^2^ for both male and female patients as well as the entire study population (N = 50).

*CT* computed tomography, *MRI* magnetic resonance imaging.

**Figure 1:**
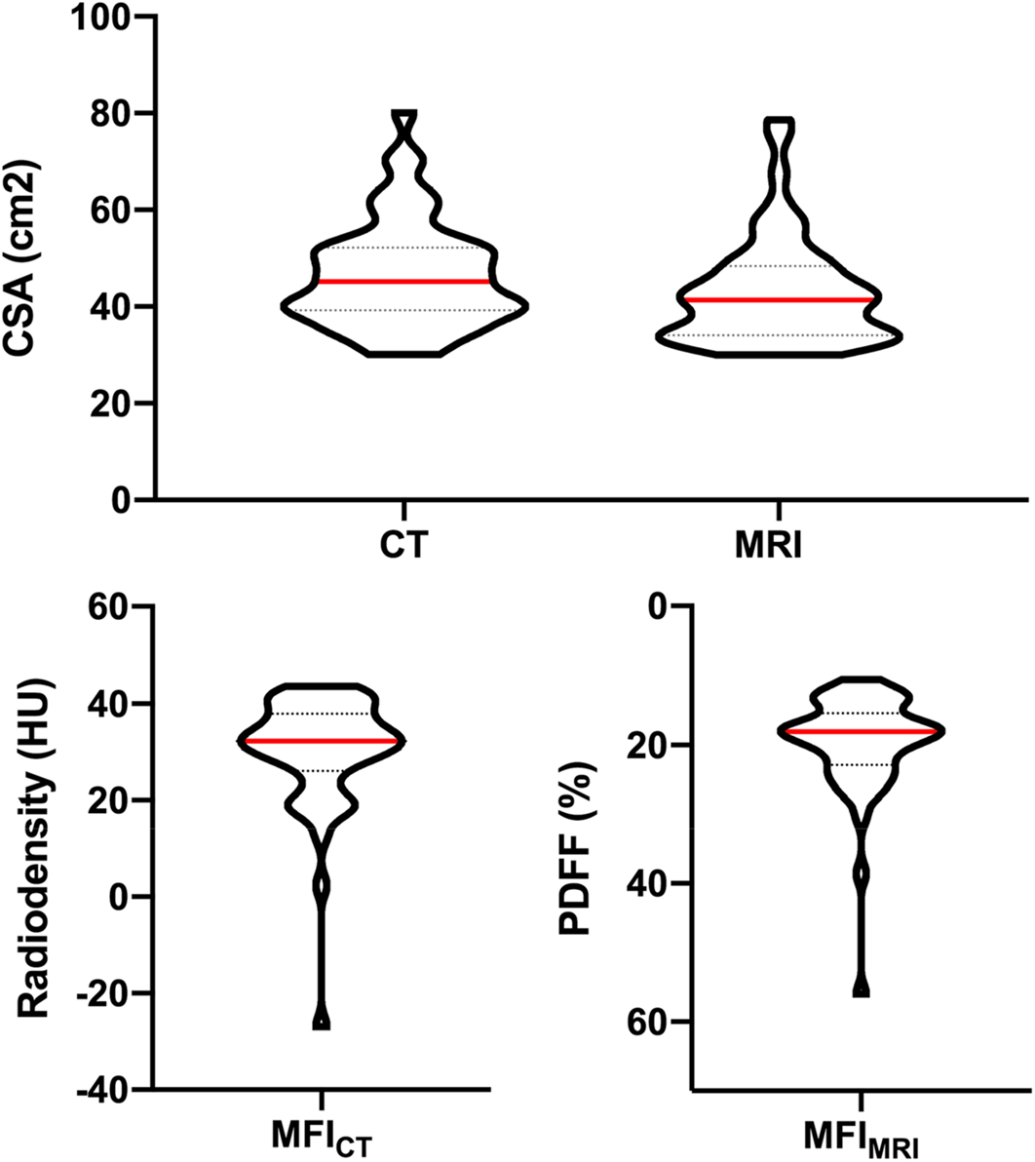
Violin plots illustrating distribution skeletal muscle area and muscle fat infiltration (MFI) measurements within the study population. All subjects underwent same day computed tomography (CT) and magnetic resonance imaging (MRI) of the chest. For each subject, axial single-slice images at the level of the superior mesenteric artery were obtained from both CT and MRI. Skeletal muscle area was defined as the bilateral cross-sectional area (CSA) of the paraspinal skeletal muscle compartment in cm^2^. For the CSA, MFI_CT_ and MFI_MRI_ were calculated as the mean radiodensity in Hounsfield units (HU) and proton density fat fraction (PDFF) in percent (%), respectively.

Mean radiation attenuation of the total CSA for the entire study population was 30 HU and ranged between -31 HU and 44 HU (Fig. 1).

No significant differences with regard to mean radiodensity of total CSA between male and female patients were observed (Table 3, P = 0.828).

**Table 3:** Assessment of CT and MRI muscle fat infiltration.

Modality	Muscle fat infiltration		All subjects (N = 50)	Male (N = 31)	Female (N = 19)	P-value
CT	Mean radiodensity	Total (HU)	30 ± 12	29 ± 15	32 ± 6	0.828
		Right (HU)	29 ± 13	28 ± 16	30 ± 7	
		Left (HU)	31 ± 11	30 ± 14	33 ± 6	
		P-value		0.675	0.070	
MRI	MRI^PDFF^	Total (%)	20 ± 8	20 ± 9	20 ± 4	0.090
		Right (%)	20 ± 8	20 ± 10	21 ± 5	
		Left (%)	20 ± 7	19 ± 9	20 ± 4	
		P-value		0.978	0.686	

CT and MRI muscle fat infiltration were assessed by mean radiodensity in Hounsfield units (HU) and proton density fat fraction (MRI^PDFF^) in percent (%), respectively. Values are given for the entire (total) paraspinal skeletal muscle compartment as well as each right and left compartment, respectively, for male and female patients as well as the entire study population (N = 50). CT: computed tomography, MRI: magnetic resonance imaging.

In MRI, the mean proton density fat fraction (MRI^PDFF^) of the total CSA for the entire study population was 20% and ranged between 11 and 56%. No significant differences between male and female patients with regard to MRI^PDFF^ was observed (P = 0.090).

### Interrelations between CT and MRI derived measurements of skeletal muscle area

**Figure 2:**
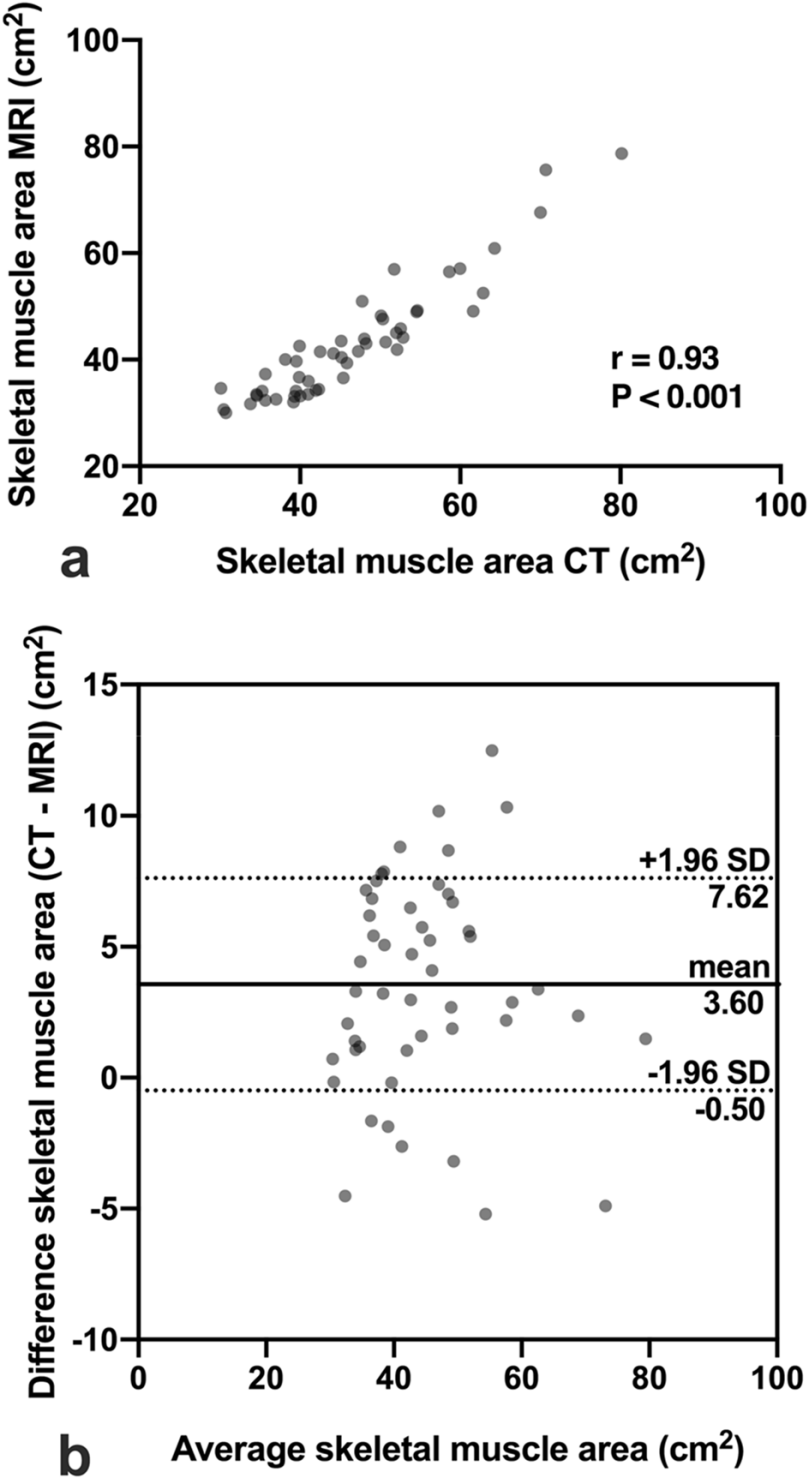
Interrelation between skeletal muscle area measurements determined by CT and MRI. Skeletal muscle area was defined as the bilateral cross-sectional area of the paraspinal skeletal muscle compartment in cm^2^ and was measured from axial single-slice images at the level of the superior mesenteric artery. Correlation analysis (**a**) and Bland–Altman analysis (**b**) were performed for intermodal comparison. CT: computed tomography, MRI: magnetic resonance imaging.

CT and MRI derived measurements of skeletal muscle area were highly correlated (r = 0.93, P < 0.001, Fig. 2a). According to Bland–Altman analysis, a bias of 3.6 cm^2^ of CT over MRI derived measurements of skeletal muscle area was identified (Fig. 2b).

**Figure 3:**
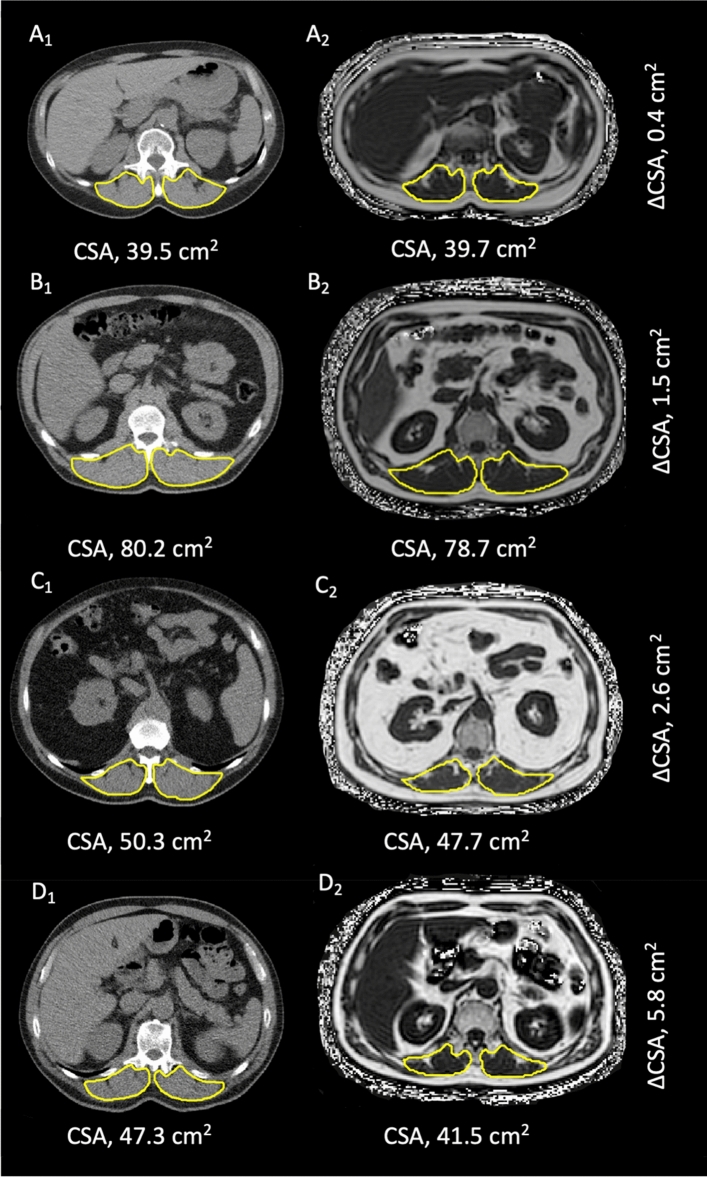
Exemplary cross-sectional images of four subjects (A_1_/A_2_, B_1_/B_2_, C_1_/C_2_, D_1_/D_2_) from the study population illustrating agreement of skeletal muscle area measurements determined by CT (A_1_–D_1_) and MRI (A_2_–D_2_). CSA: cross-sectional area of the paraspinal skeletal muscle compartment, CT: computed tomography, MRI: magnetic resonance imaging, ΔCSA: intra-individual difference between CT and MRI derived skeletal muscle area measurements. Single-slice images were derived from image datasets at the level of the offspring of the superior mesenteric artery. Minimal intra-individual differences in caption of this landmark between respective imaging modalities (CT/MRI) are unavoidable due to differences in breathing as well as patient positioning.

Exemplary images of CSA in intraindividual, intermodal comparison of patients from the study population are illustrated in Fig. 3**.**

### Interrelations between CT and MRI derived measurements of muscle fat content

**Figure 4:**
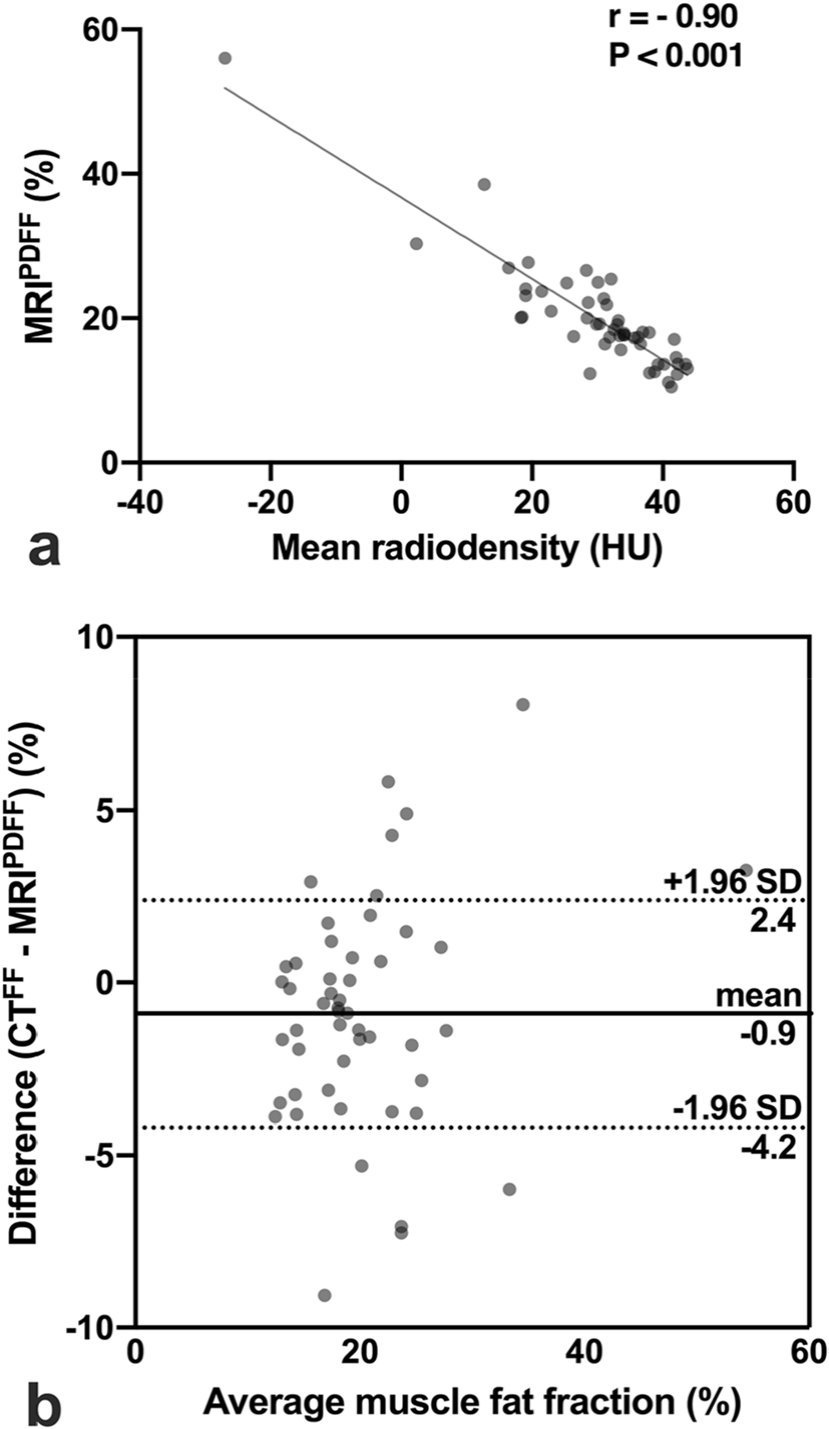
Interrelation of muscle fat infiltration measurements. CT and MRI muscle fat infiltration were assessed by mean radiodensity in Hounsfield units (HU) and proton density fat fraction (MRI^PDFF^) in percent (%), respectively. Based on the correlation analysis (**a**), a linear regression model was fit to calculate CT derived fat fraction (CT^FF^). CT^FF^ and MRI^PDFF^ were compared with the Bland–Altman method (**b**). CT: computed tomography, MRI: magnetic resonance imaging.

Measurements for determination of muscle fat infiltration (MFI) in CT and MRI were highly correlated (r = − 0.90, P < 0.001, Fig. 4a). Based on this interrelation, a linear regression model was fit to calculate CT derived fat fraction (CT^FF^) from mean radiodensity of the CSA of the total paraspinal skeletal muscle compartment based on the corresponding MRI^PDFF^ values. CT^FF^ was defined by the following equation:

$$CT^{{FF}} (\% )\, = \, - 0.562{\text{ }}*{\text{ }}mean{\text{ }}radiodensity{\text{ (}}HU{\text{)}}\, + \,36.71$$

Mean CT^DFF^ was 21% and ranged between 13 and 53%. Bland–Altman analysis demonstrated a bias of -0.9% of CT^FF^ over corresponding MRI^PDFF^ values (Fig. 4b).

**Figure 5:**
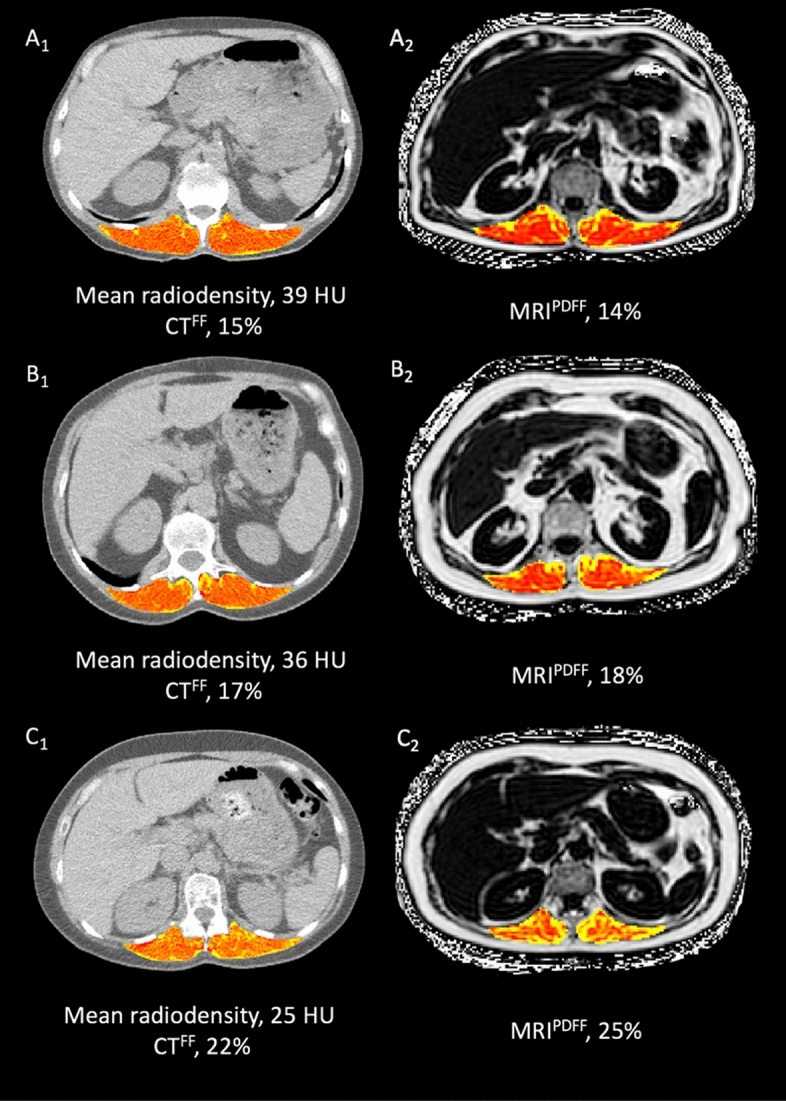
Exemplary cross-sectional images of three subjects (A_1_/A_2_, B_1_/B_2_, C_1_/C_2_) from the study population illustrating agreement of CT and MRI derived muscle fat infiltration measurements. CT and MRI muscle fat infiltration were calculated as mean radiodensity in Hounsfield units (HU) and proton density fat fraction (MRI^PDFF^) in percent (%), respectively. Based on the correlation between CT and MRI muscle fat infiltration, a linear regression model was fit to calculate CT derived fat fraction (CT^FF^) from mean radiodensity based on the corresponding MRI^PDFF^ values. *CT* computed tomography, *MRI* magnetic resonance imaging.

Exemplary images of CT and MRI derived MFI measurements in an intraindividual comparison are illustrated in Fig. 5.

## Discussion

The purpose of this prospective study was to evaluate the interrelation between CT and MRI derived imaging biomarkers of sarcopenia. As the key finding, imaging biomarkers of both skeletal muscle mass and quality were highly correlated in an intra-individual, intermodal comparison. The provided results allowed for development of a linear regression model to directly estimate skeletal muscle fat content from CT based on the corresponding MRI^PDFF^ values, which in the future may help to improve clarity of CT derived determination of skeletal muscle fat content as an indicator of skeletal muscle quality.

Objective and reliable assessment of skeletal muscles is mandatory to make the diagnosis of sarcopenia^1,10^. Both CT and MRI are used for several indications in clinical routine. As demonstrated in previous studies, clinical CT and MRI may be used beyond their primary diagnostic purposes to opportunistically obtain measurements of body composition, including skeletal muscle mass and quality^8,11–13,17^. This approach allows for obtainment of additional, potentially relevant prognostic information from routine diagnostic imaging, avoiding additional examinations, and may enhance the feasibility of sarcopenia assessment in clinical routine. However, to warrant wide applicability of opportunistic body composition assessment from diagnostic imaging, the agreement between CT and MRI derived measurements needs to be determined.

Two previous studies investigated the agreement between CT and MRI derived measurements of skeletal muscle area^18,19^. Although these studies indicated that estimates of skeletal muscle mass in CT and MRI were highly correlated, the large time span of up to 101 days between the respective examinations, which were used for intra-individual comparison in these studies, may be considered an important confounder. Factors such as aging, physical inactivity, or wasting due to acute and chronic diseases are known to induce decline of skeletal muscle mass and, as demonstrated very recently, substantial changes in skeletal muscle area may be observed within a few days of physical inactivity^20,21^. Therefore, investigating the intermodal agreement of skeletal muscle area measurements in a same-day setting, our results may be considered to substantiate and further validate insights from these previous studies. In accordance with former reports, we observed that when compared directly, measured skeletal muscle area tends to be systematically larger in CT compared to MRI^18,19^. It is likely that at least to some extent this observation may be related to differences in patient positioning, which would be in agreement with another study investigating the interrelation of musculoskeletal measurements between CT and MRI^22^. While in MRI, patients were positioned with arms along the body, chest CT scans were performed with arms raised above the head in our study, as it is common practice. Possibly, consecutive alterations in trunk positioning may have influenced area-based measurements of the paraspinal skeletal muscle compartment. Also, MRI examinations are typically performed in expiration, while CT investigations are mostly performed in inspiration. It is unclear if other anatomical landmarks, particularly the lumbar vertebral levels, may be less affected by confounding factors such as body positioning or breathing. Future studies are warranted to eventually solve this issue.

The recently updated European consensus guidelines on definition and diagnosis of sarcopenia stress the particular role of reduced muscle function as a key element of sarcopenia, as it was demonstrated to better predict adverse outcomes in various conditions compared to exclusive evaluation of muscle mass^1,10,23,24^. A central component of muscle function is strength, which again was shown to be strongly associated with MRI^PDFF^ as a measure of skeletal muscle fat content and thereby indicator of muscle quality^9^. Skeletal muscle fat content refers to the composition as well as the micro- and macroscopic elements of tissue architecture—which can be assessed from both CT and MRI—and was demonstrated to reveal prognostic information in various diseases^11–13,16,25^. Measurements of skeletal muscle fat content as indicators of muscle quality are therefore considered promising new imaging biomarkers of sarcopenia, which in the future are expected to support treatment decision and response assessment^1^. Accordingly, a particular focus of our study was to evaluate the agreement between CT and MRI derived biomarkers of muscle quality, which to our knowledge has not been done so far. The high correlation between muscle radiodensity in CT and corresponding MRI^PDFF^ allowed us to calculate a linear regression model to directly estimate the degree of myosteatosis from HU values. This approach provides CT^FF^ as a CT-based measure of skeletal muscle fat content on a ratio scale, which may improve clarity and thereby may enhance not only applicability in clinical routine but also may be of particular interest for larger cohort studies.

Several approaches with different anatomical landmarks for assessment of skeletal muscles from axial single-slice images have been proposed in literature. The most common are determination of the skeletal muscle index, defined as the entire skeletal muscle area at the level of the third lumbar vertebra normalized for body height as well as the psoas skeletal muscle area, assessed at the level of the third or fourth lumbar vertebra^26^. However, those landmarks are frequently not captured within several imaging protocols, for example those required for liver imaging, although body composition assessment may be particularly relevant in patients undergoing these examinations^11^. The concept of opportunistic imaging precludes extension of imaging window for sole assessment of body composition. Therefore, some previous studies investigated the interrelation of different vertebral levels with skeletal muscle mass and proposed alternative landmarks, such as the SMA level^6,7,27^. We decided to perform skeletal muscle measurements at the SMA level, since it is regularly captured within the most common imaging protocols of the abdomen and chest, is clearly defined and can be easily determined from the axial plane, and finally was suggested to reveal prognostic information in various chronic and malignant diseases^11,13^.

We are aware of several limitations of our study. First, it would have been interesting to study the variability of skeletal muscle measurements among different MRI sequences. However, a primary goal of this study was to investigate the intermodal agreement of imaging based biomarkers of muscle quality as a surrogate of muscle strength, which in MRI can be reliably assessed using MRI^PDFF^^9^. Previous studies demonstrated a high agreement of different MRI sequences for determination of skeletal muscle mass^19^ and indicated that measurements of skeletal muscle quantity from PDFF are highly reliable and repeatable^9,25^. Therefore, in MRI we decided to perform muscle measurements from PDFF maps. Future studies could eventually determine its role compared to other MRI sequences. Secondly, in clinical routine CT scans are frequently performed after administration of intravenous contrast agents and are acquired in different scan phases, depending on the primary question of the examination. As in this study healthy participants of a cancer screening program were investigated, the study setting precluded administration of intravenous contrast agents. A previous study indicated that with respect to measurements of skeletal muscle fat content, distinct differences between phases of CT scans can be observed^28^. However, as the total magnitude of these differences appeared to be very small, the clinical impact of these findings is not clear and should be investigated in future studies. The question if muscle measurements may vary across different imaging platforms and scanning parameters in CT and MRI is unresolved to date. To account for this, in our study all CT and MRI scans were performed at the same respective unit with fixed scanning parameters. A recent report indicated a low level of bias across field strengths and imaging platforms for determination of fat content using MRI^PDFF^^29^. However, as this study used MRI^PDFF^ to determine vertebral bone marrow fat content, it is not known if those results are directly transferable to myosteatosis measurements. To the best of our knowledge, the bias among vendors and scanners for determination of fat content from CT has not been studied so far. This issue should be clarified in future studies.

To conclude, our results indicate a high agreement of imaging-derived biomarkers of muscle quantity and quality between CT and MRI in healthy subjects, indicating that both modalities may be used interchangeably for skeletal muscle assessment. Therefore, this study warrants larger and prospective studies to assess intermodal agreement, particularly in sarcopenia patients. Regarding CT-derived measurements of muscle quality, the provided results allowed for development of a linear regression to directly estimate skeletal muscle fat content from CT based on the corresponding MRI^PDFF^ values, which in the future may enhance clarity of muscle quality measurements in CT and may also be particularly relevant for body composition analysis in larger cohort studies.

## Data Availability

The datasets generated during and/or analyzed during the current study are available from the corresponding author on reasonable request.
